# Hexachlorophene Is a Potent KCNQ1/KCNE1 Potassium Channel Activator Which Rescues LQTs Mutants

**DOI:** 10.1371/journal.pone.0051820

**Published:** 2012-12-12

**Authors:** Yueming Zheng, Xuejing Zhu, Pingzheng Zhou, Xi Lan, Haiyan Xu, Min Li, Zhaobing Gao

**Affiliations:** 1 Key Laboratory of Receptor Research, Shanghai Institute of Materia Medica, Chinese Academy of Sciences, Shanghai, China; 2 Department of Neuroscience, High Throughput Biology Center and Johns Hopkins Ion Channel Center, School of Medicine, Johns Hopkins University, Baltimore, Maryland, United States of America; Sackler Medical School, Tel Aviv University, Israel

## Abstract

The voltage-gated KCNQ1 potassium channel is expressed in cardiac tissues, and coassembly of KCNQ1 with an auxiliary KCNE1 subunit mediates a slowly activating current that accelerates the repolarization of action potential in cardiomyocytes. Mutations of *KCNQ1* genes that result in reduction or loss of channel activity cause prolongation of repolarization during action potential, thereby causing long QT syndrome (LQTs). Small molecule activators of KCNQ1/KCNE1 are useful both for understanding the mechanism of the complex activity and for developing therapeutics for LQTs. In this study we report that hexachlorophene (HCP), the active component of the topical anti-infective prescription drug pHisoHex, is a KCNQ1/KCNE1 activator. HCP potently increases the current amplitude of KCNQ1/KCNE1 expressed by stabilizing the channel in an open state with an EC_50_ of 4.61±1.29 μM. Further studies in cardiomyocytes showed that HCP significantly shortens the action potential duration at 1 μM. In addition, HCP is capable of rescuing the loss of function of the LQTs mutants caused by either impaired activation gating or phosphatidylinositol-4,5-bisphosphate (PIP2) binding affinity. Our results indicate HCP is a novel KCNQ1/KCNE1 activator and may be a useful tool compound for the development of LQTs therapeutics.

## Introduction

KCNQ (or Kv7) channels are voltage-gated potassium channels. They mediate sub-threshold, noninactivating voltage-gated potassium currents that have important roles in controlling membrane excitability [1]. Of the five known isoforms, KCNQ1–5, KCNQ1 is the only one predominantly expressed in heart. KCNQ1 is the pore forming subunit, tetrameric KCNQ1 complexes give rise to functional channels. In native cells such as cardiomyocytes, KCNQ1 coassembles with a non-conductive accessory KCNE1 subunit, a small single transmembrane protein encoded by *KCNE1* gene. The heteromultimeric KCNQ1/KCNE1 was proposed to mediate a slowly activating current that accelerates the repolarization of action potential in cardiac tissues, also known as IKs [2], [3].

Loss-of-function mutations in KCNQ1 lead to long QT syndrome (LQTs), a severe arrhythmia characterized by an abnormality in cardiac repolarization leading to prolonged QT interval [4]–[6]. The severity of LQTs varies from syncope to sudden death. LQTs can be either congenital or acquired. More than 50% congenital LQTs cases and 90% LQTs occurring during exercise are linked to mutations in the *KCNQ1* gene. Genetic studies of LQT patients have identified at least 113 KCNQ1 mutations, including missense (86/113), nonsense (6/113), deletion (13/113), frame shift (1/113) and splice (7/113) mutations [7]. Potentiation of the KCNQ1 channel by small molecule activators is thought to be a potential and attractive strategy to treat LQTs.

Recently, a number of activators of KCNQ channels have been reported [8]–[12]. However, activators for KCNQ1 are still rare and few are effective on the physiologically relevant KCNQ1/KCNE1 complex [13]. Two known examples include R-L3 and zinc pyrithione (ZnPy) [13], [14]. Both potentiate homomeric KCNQ1 channel but lack sensitivity to the KCNQ1/KCNE1 complex. To date, only three small molecule activators for the KCNQ1/KCNE1 complex have been identified. They are mefenamic acid (MFA), DIDS and phenylboronic acid (PBA). Initially, MFA and DIDS were identified as chloride channel blockers [15]. These compounds strongly potentiate the KCNQ1/KCNE1 but exhibit little effect on homomeric KCNQ1. In contrast, PBA, an aromatic derivative of boronic acids, potentiates both the homomeric KCNQ1 and the KCNQ1/KCNE1 complex with millimolar effective concentration [16].

We screened a collection of 1,280 drugs or drug candidates against homomeric KCNQ channels and identified HCP as one of active compounds. HCP, also known as Nabac, is the active component of pHisoHex, a topical anti-infective prescription drug [17]. We found that both the homomeric KCNQ1 and the KCNQ1/KCNE1 complex were sensitive to HCP at micromolar concentrations and the effect on the KCNQ1/KCNE1 complex is much more potent than that on homomeric KCNQ1. Further studies showed that HCP was effective in cardiomyocytes and was capable of rescuing the LQTs KCNQ1 mutants. Taken together, our study indicates that HCP as an effective KCNQ1/KCNE1 activator.

## Materials and Methods

### Cell culture and transfection

CHO cells were grown in 50/50 DMEM/F-12 (Gibco) with 10% fetal bovine serum (FBS), and 2 mM L-glutamine (Invitrogen). To express the channels and mutants, cells were split at 24 h before transfection, plated in 60-mm dishes, and transfected with Lipofectamine 2000^TM^ reagent (Invitrogen), according to the manufacturer’s instructions. A GFP cDNA (Amaxa, Gaithersburg, MD) was cotransfected to identify the transfected cells by fluorescence microscopy.

### cDNA and mutagenesis

The KCNQ1 to KCNQ4 and KCNE1 cDNA were gifts from Drs. T. Jentsch (Zentrum für Molekulare Neurobiologie, Hamburg), D. Makinnon (State University of New York, Stony Brook), M. Sanguinetti (University of Utah), M. Shapiro (University of Texas Health Science Center, San Antonio) and V. Vardanyan (Universität Hamburg), respectively. Point mutations were introduced by using the QuikChange II site-directed mutagenesis kit (Stratagene, La Jolla, CA), and verified by DNA sequencing.

### FluxOR thallium assay

CHO cells stably expressing the rat KCNQ2 were routinely cultured in DMEM/F12 medium, supplemented with 10% FBS and 500 μg/mL G418. The FluxOR thallium assay protocol was the manufacturer’s protocol. CHO-KCNQ2 cells were seeded in wells of 96-well plates at ∼10,000 cells/well and grown until 80–90% confluence at 37°C in a 5% CO_2_ incubator. The medium was removed the following day and 80 μL of FluxOR loading buffer was added to each well for 90 min at room temperature (RT) in darkness. After removing the loading buffer, 100 μL/well of assay buffer and 20 μL/well of 7X control/test compound were added to cells at RT in darkness. Compounds to be tested were prepared using assay buffer; controls were assay buffer (EC_0_), EC_50_ of Retigabine and EC_100_ of ztz240. After 30 min, cell plates were loaded on FDSS. After 10 seconds of recording, 20 μL/well of stimulus buffer was added. The plates were read every second for 110 s. The stimulus buffer contained 1.30 mM K2SO4 and 9.80 mM Tl_2_SO_4_. The △potentiation% ((R_test_-R_control_)/(R_control_-R_buffer_)*100%) was calculated for each well using the 35 second fluorescence ratio. To identify compounds with potentiation activity on KCNQ channels, a thallium flux assay was developed and used to screen a Microsource™ library of 1,280 compounds at 10 μM final concentration. In the pilot screening, HCP exhibited strong potentiation on the fluorescence signal of KCNQ2.

### Electrophysiology recording in CHO cells

To record current of the expressed KCNQ channels in CHO cells, standard whole-cell recording was used. Pipettes were pulled from borosilicate glass capillaries (TW150-4, World Precision Instruments). When filled with the intracellular solution, the pipettes have resistances of 3–5 megaohms. During the recording, constant perfusion of extracellular solution was maintained using a BPS perfusion system (ALA Scientific Instruments). Pipette solution contained (in mM): 145 KCl, 1 MgCl_2_, 5 EGTA, 10 HEPES and 5 MgATP (pH 7.3); extracellular solution contained (in mM): 140 NaCl, 3 KCl, 2 CaCl_2_, 1.5 MgCl_2_, 10 HEPES and 10 glucose (pH 7.4). Current and voltage were recorded using an Axopatch-200B amplifier, filtered at 2 kHz, and digitized using a DigiData 1440A with pClamp 10.2 software (Axon Instruments). Series resistance compensation was also used and set to 60–80%.

### Isolation of cardiomyocytes, IKs and action potential recording

All animal procedures were performed in accordance with the National Institute of Heath Guide for the Care and Use of Laboratory Animals, under protocols approved and strictly followed by the Institutional Animal Care and Use Committees (IACUC). The IACUC checked all protocols and approved this study. Single myocytes were isolated from the left ventricle of adult guinea pig in a Langerdorff perfusion system as previously described [18]. The hearts were removed quickly via midline thoracotomy and perfused with a Ca^2+^-free Tyrode’s solution containing collagenase (6 mg/ml) and protease (0.1 mg/ml) for ∼5–6 min. Then switched to Kraft-Bruhe (KB) solution perfusion 5 minutes and the ventricles were minced and gently triturated to single cells. The cells were stored at 4°C in KB solution until use. KB solution contained the following (in mM): L-glutamic acid 50, KOH 80, KCl 40, MgSO_4_ 3, KH_2_PO_4_ 25, HEPES 10, EGTA 1, taurine 20 and glucose 10 (pH 7.4). To record the IKs, myocytes were perfused with Na^+^ free solution containing (mM): N-methyl-D-glucamine 132 (for sodium ion replacement), CaCl_2_ 1.0, MgCl_2_ 1.0, HEPES 10, Glucose 5, lanthanum chloride 0.05 to block IKr and nifedipine 0.005 to block L-type calcium current (pH = 7.4 with HCl). The intracellular solution contains (mM): KCl 120, KH_2_PO_4_ 10, MgSO_4_ 1, EGTA 5, HEPES 5 (pH = 7.2 with KOH). The action potential was recorded at 35–37°C. To induce action potentials, a 10-ms depolarizing pulse was applied at a frequency of 0.16 Hz. Extracellular solution contains (in mM): NaCl 135, KCl 5.4, CaCl_2_ 1.8, MgCl_2_ 1, HEPES 5 and glucose 10 (pH 7.4); intracellular solution contained (in mM): potassium aspartate 120, KCl 20, MgSO_4_ 1, Na_2_ATP 4 and HEPES 10 (pH 7.2).

### Data analysis

Patch clamp data were processed using Clampfit 10.2 (Molecular Devices, Sunnyvale, CA) and then analyzed in GraphPad Prism 5 (GraphPad Software, San Diego, CA). Voltage-dependent activation curves were fitted with the Boltzmann equation, *G* = *G*_min_+(*G*_max_-*G*_min_)/(1+exp(*V*-*V*_1/2_)/S), where *G*_max_ is the maximum conductance, *G*_min_ is the minimum conductance, *V*_1/2_ is the voltage for reaching 50% of maximum conductance, and S is the slope factor. Dose-response curves were fitted with the Hill equation, E = E_max_/(1-(EC_50_/C)P), where EC_50_ is the drug concentration producing half of the maximum response, and P is the Hill coefficient. Data are presented as means ± SD. Significance was estimated using unpaired two-tailed Student’s t tests. An effect was considered significant if *p*<0.05.

## Results

### Subtype selectivity of HCP

**Figure 1:**
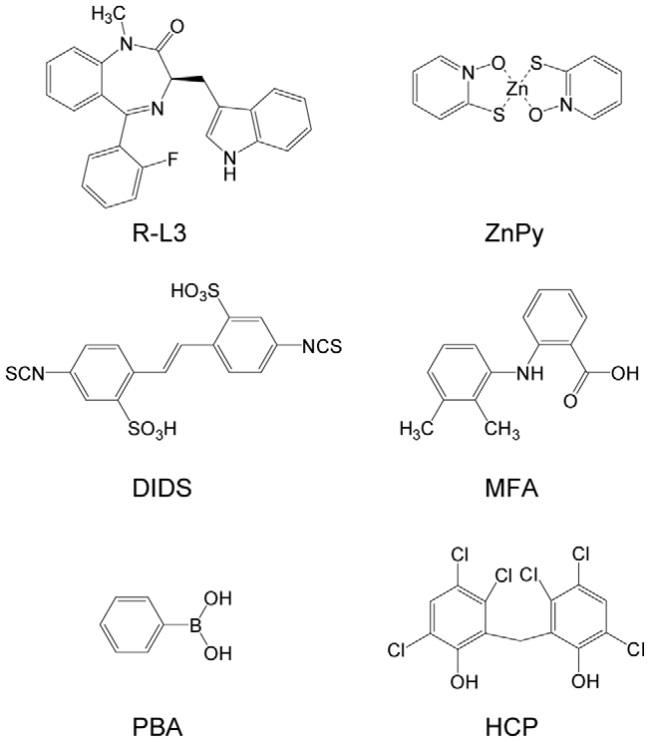
Chemical structures of reported KCNQ1 or KCNQ1/KCNE1 activators and HCP. R-L3 and ZnPy potentiate the KCNQ1 but not the KCNQ1/KCNE1 complex. DIDS and MFA strongly potentiate the KCNQ1/KCNE1 complex but exhibit little effect on the homomeric KCNQ1. PBA potentiates both the KCNQ1 and the KCNQ1/KCNE1 complex.

**Figure 2:**
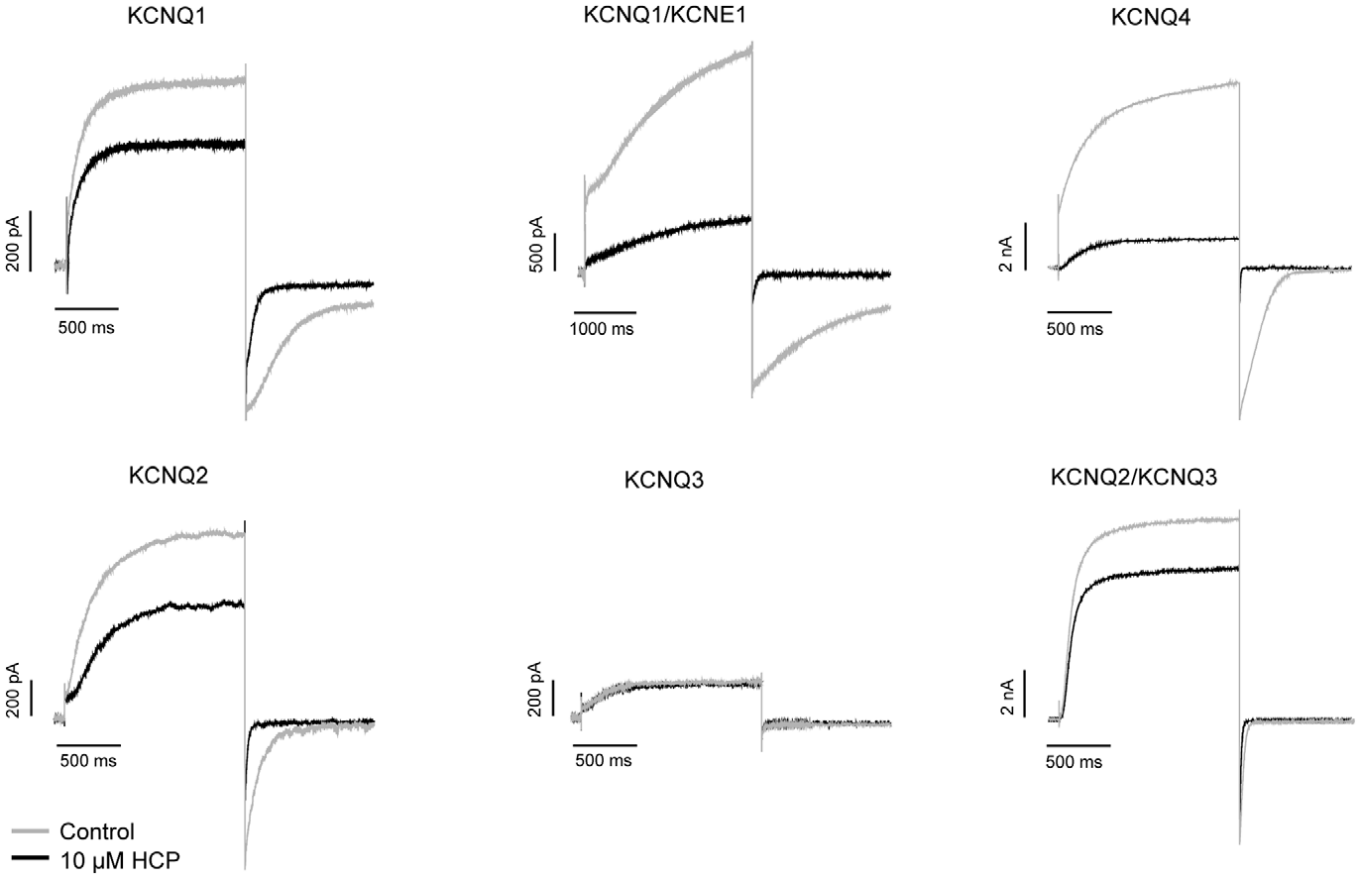
Subtype selectivity of HCP. For all tested channels, the holding potential was set at −100 mV and the currents were elicited by a depolarization step to −10 mV.

Structurally, HCP is distinct from all previously reported KCNQ1 or KCNQ1/KCNE1 activators (Fig. 1). To obtain a better understanding for HCP activity on KCNQ channels, we examined its effects on KCNQ1, KCNQ1/KCNE1, KCNQ2, KCNQ3, KCNQ2/3 and KCNQ4 isoforms by electrophysiology. To elicit the currents, a fixed −10 mV depolarization was used for all tested channels. Table 1 summarizes 10 μM HCP potentiation on the indicated outward currents. HCP potentiates all tested channels except KCNQ3 (Fig. 2). Noticeably, HCP significantly potentiates both KCNQ1 and the KCNQ1/KCNE1 complex. The *I*/*I*_0_ values were 1.48±0.13 and 4.47±0.70, respectively. The effects of HCP on *G*-*V* curves of these subtypes were examined and summarized in Table 1. The unique subtype selectivity, *i.e*., strong potentiation on both KCNQ1 and KCNQ1/KCNE1, supports HCP is different from previous reported KCNQ1 activators.

**Table 1:** Effects of HCP on the outward current of different KCNQ channels.

Channel Subtype	*I*/*I*_0_	Δ*V*_1/2_	n
KCNQ1	1.48±0.13*	−23.37±1.60*	7
KCNQ1/KCNE1	4.47±0.70*	−66.05±6.15*	7
KCNQ2	1.65±0.22*	−40.05±1.91*	4
KCNQ3	1.02±0.03	NA	3
KCNQ2/KCNQ3	1.25±0.13*	−27.66±2.13*	4
KCNQ4	6.87±2.27*	−68.77±9.62*	5

*I*/*I*_0_ indicates the compounds’ effects on amplitude of outward current of KCNQ channels. *I*_0_: the amplitude of outward current in the absence of the compound. *I*: the amplitude of outward current in the presence of the compound. To calculate *I*/*I*_0_, the depolarization is +50 mV for KCNQ1/KCNE1 and is −10 mV for all other subtypes. Δ*V*_1/2_ indicates the shifting of *G*-*V* curve. To calculate *V*_1/2_, the tail current elicited by followed −120 mV was measured. The concentration of HCP was 10 μM. For KCNQ3, the Δ*V*_1/2_ was not available (NA) because the KCNQ3 currents at low voltage potentials were too small to be measured accurately. * *p*<0.05 versus control.

### Potentiation of the KCNQ1 by HCP

**Figure 3:**
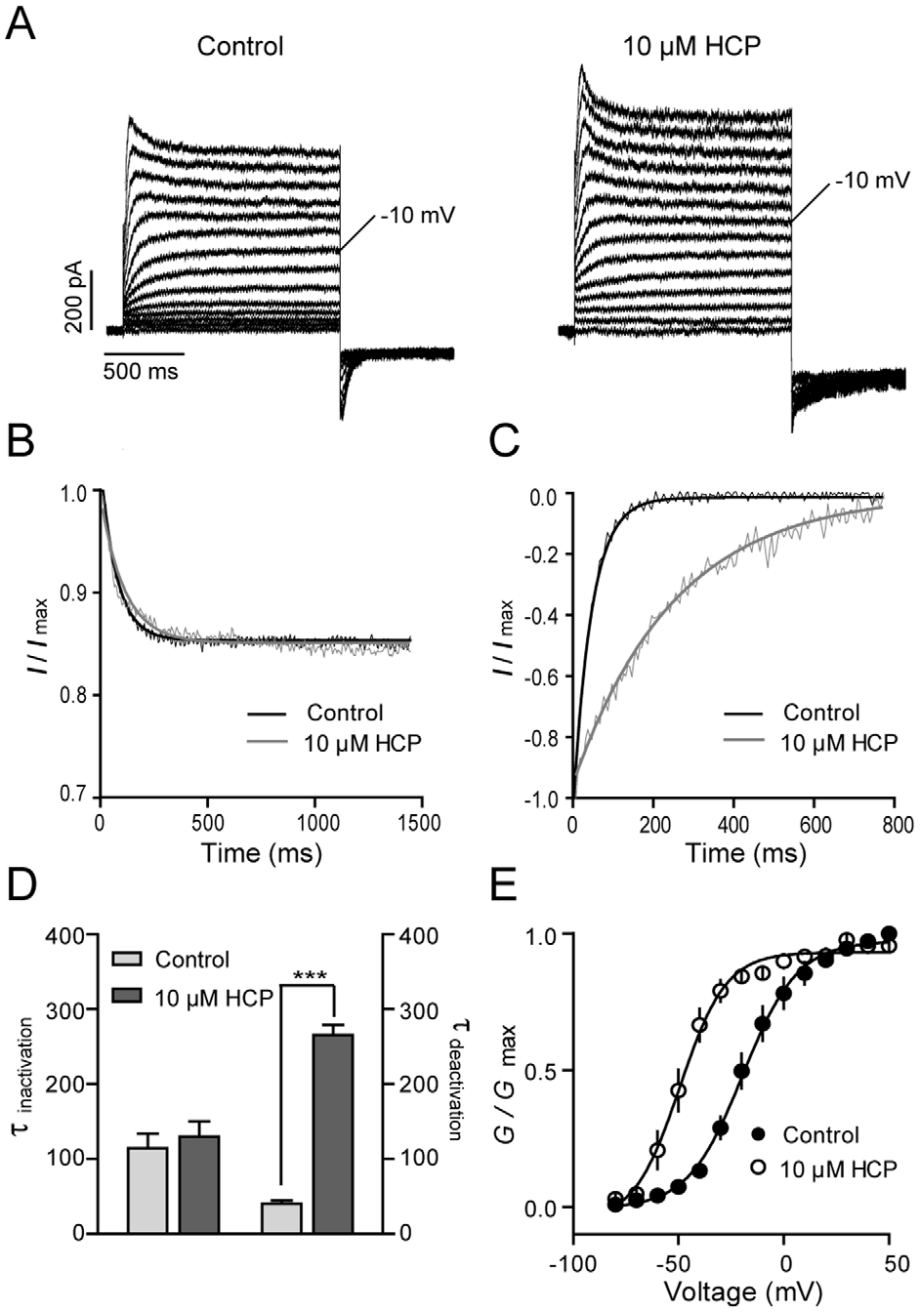
Effects of HCP on the homomeric KCNQ1 channel. **A.** Representative traces of the homomeric KCNQ1 channel in the absence and presence of 10 μM HCP. **B**. The normalized inactivation phase from full traces in the absence (black line) and presence (gray line) of 10 μM HCP. The testing depolarization is +50 mV. **C**. The normalized tail current from full traces in the absence (black line) and presence (gray line) of 10 μM HCP. The tailed currents were elicited by a hypolarization step to −120 mV followed the +50 mV depolarization. **D**. Histogram summarized the effects of 10 μM HCP on inactivation and deactivation of KCNQ1 (n≥3, ***p<0.001 versus control). **E**. *G*-*V* curves in the absence (filled circle) and presence (open circle) of 10 μM HCP (n≥3).

The homomeric KCNQ1 mediates a characteristic outward current with detectable inactivation (Fig. 3A). In the presence of 10 μM HCP, steady-state outward currents at different depolarizing voltages were greatly potentiated. The potentiation on the KCNQ1 channel mediated by previous reported KCNQ1 activators, such as ZnPy and R-L3, involves a hyperpolarizing shift of *V*_1/2_. To determine the HCP effects on the homomeric KCNQ1, we examined the *G*-*V* curves of the KCNQ1 in the absence and presence of 10 μM HCP. Before application of HCP, the *V*_1/2_ value was −19.43±1.39 mV (Fig. 3E). After application of HCP, the *V*_1/2_ value was shifted to −49.35±1.93 mV with a left-shifting around 30 mV. Inactivation is a distinctive feature for KCNQ1. The inhibition of inactivation by activators, such as ZnPy, is thought to be a contributing factor for an overall increase of current amplitude [13]. We analyzed the inactivation kinetics at different depolarization steps and found no detectable difference before and after application of HCP (Fig. 3B). Hence, HCP increases overall conductance of the KCNQ1 by left-shifting the *G*-*V* curve but not affecting inactivation. In addition, HCP also induced delaying of deactivation in hyperpolarizing voltages (Fig. 3C). In the presence of 10 μM HCP, time constant of deactivation was slowed from 40.05±7.52 ms to 265.51±23.23 ms (n = 3, *p*<0.01). The reduction of deactivation rate is consistent with the overall increase of current amplitude or *G*_max_.

### HCP potentiates the KCNQ1/KCNE1 complex

**Figure 4:**
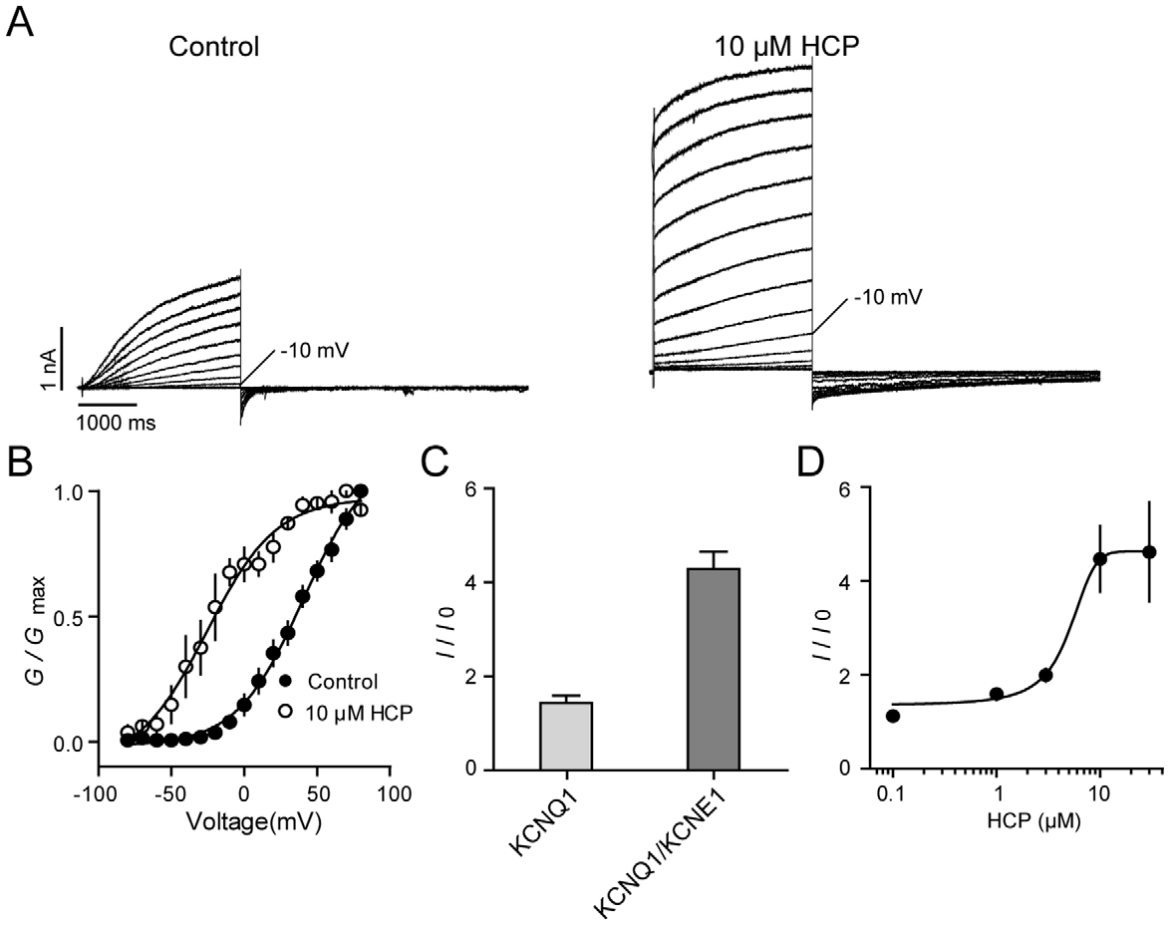
Effects of HCP on the KCNQ1/KCNE1 complex. **A**. Representative traces of the KCNQ1/KCNE1 complex in the absence and presence of 10 μM HCP. The holding potential was set at −100 mV. The currents were elicited by a series of depolarization from −90 mV to +80 mV. **B**. *G*-*V* curves of the KCNQ1/KCNE1 complex. **C**. Potentiation of 10 μM HCP on outward currents of the KCNQ1/KCNE1. *I*/*I*_0_, *I*, and *I*_0_ are same as being referred in Table 1. **D**. Dose-response curve of HCP on the KCNQ1/KCNE1 complex. n≥3 of each data point.

To examine HCP modulation on KCNQ1/KCNE1 complex, we co-expressed the cDNAs of KCNQ1 and KCNE1 (1:1) in CHO cells. Consistent with previous reports, the effects of KCNE1 on KCNQ1 include increase in overall current amplitude, slowing of the activation and deactivation kinetics, and removal of inactivation (Fig. 4A). We tested HCP effects at −10 mV, which is the same potential to elicit the KCNQ1 channel. We found that in the presence of 10 μM HCP, after around 120 seconds, a large instantaneous current became evident. Finally, the outward current was potentiated around 4.47±0.70 fold, which is much higher than that of the KCNQ1 (Fig. 4C). Similar to effects on KCNQ1, HCP also slowed the deactivation of the KCNQ1/KCNE1 complex (Fig. 4A) and left-shifted the *G-V* curve (Fig. 4B). Analysis of the dose-response curve of HCP on the KCNQ1/KCNE1 complex revealed an EC_50_ value of 4.61±1.29 μM.

### HCP decreases the action potential duration

**Figure 5:**
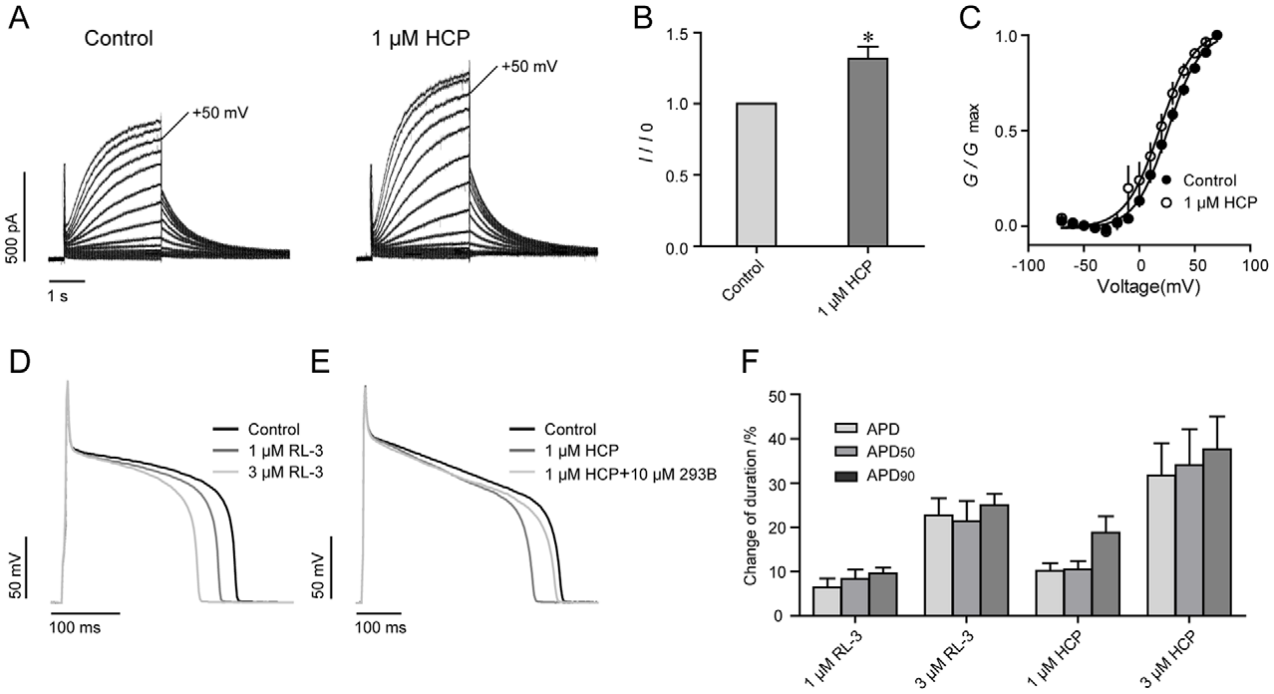
Effects of HCP on the action potential recorded in cardiomyocytes. **A**. Representative traces of IKs before and after application of 1μM HCP. To elicit the IKs, a series of depolarization steps from −70 mV to +70 mV in 10 mV increments were applied. **B**. Effects of 1 μM HCP on amplitude of IKs measured at +50 mV. * *p*<0.05 versus control. **C**. *G*-*V* curves of IKs. **D**. Effects of R-L3 on action potentials. **E**. Effects of HCP on action potentials. Chromonal 293B (10 μM) was co-applied with 1 μM HCP after steady state inhibition. **F**. Histograms show the change of action potential duration after application of R-L3 and HCP.

Because HCP potentiates the KCNQ1/KCNE1 complex, we tested its effects on IKs in acutely isolated guinea pig cardiomyocytes. We found after application of 1 μM HCP, the IKs was increased 1.34±0.15 fold (n = 4) at +50 mV measured potential. The *G*-*V* curve was left shifted −7.13±2.44 mV (n = 4) (Fig. 5A, B & C). The effects of HCP on action potential were further examined. The action potential was elicited on isolated guinea pig ventricular myocytes by current injection. Upon application of 1 μM HCP, the action potential duration (APD) was shortened by 10.24±1.71%. Consistently, the APD_90_ (action potential duration at 90% repolarization) and APD_50_ (action potential duration at 50% repolarization) were shortened by 10.58±1.88% and 18.82±3.65%, respectively (n = 7) (Fig. 5E & F). The inhibitory effects can not restore in five minutes after removal of the compound but can be reversed by Choromonal 293B, a blocker of IKs. Choromonal 293B at 10 μM alone prolonged the APD around 30.04±3.15% (n = 4). After co-application of 10 μM Choromonal 293B, the action potential duration was restored (n = 4) (Fig. 5E). Accordingly, the effects of 1 μM HCP on APD was reduced to 5.43±1.54% (n = 4). HCP at 3 μM effects caused further reduction of action potential duration. The change of APD, APD_90_ and APD_50_ were 31.71±7.3, 34.11±8.09 and 37.65±7.36, respectively (n = 9). R-L3, a reported IKs activator which shortens APD effectively, was tested as a positive control [14]. In our system, 1 μM and 3 μM R-L3 significantly deceased the APD_50_ about 9.59±1.29% (n = 3) and 25.30±2.29% (n = 3) (Fig. 5D), which were consistent with the previous study. Thus, these data are consistent with the notion that HCP acts on the native KCNQ channels.

### HCP rescues LQTs mutants

**Figure 6:**
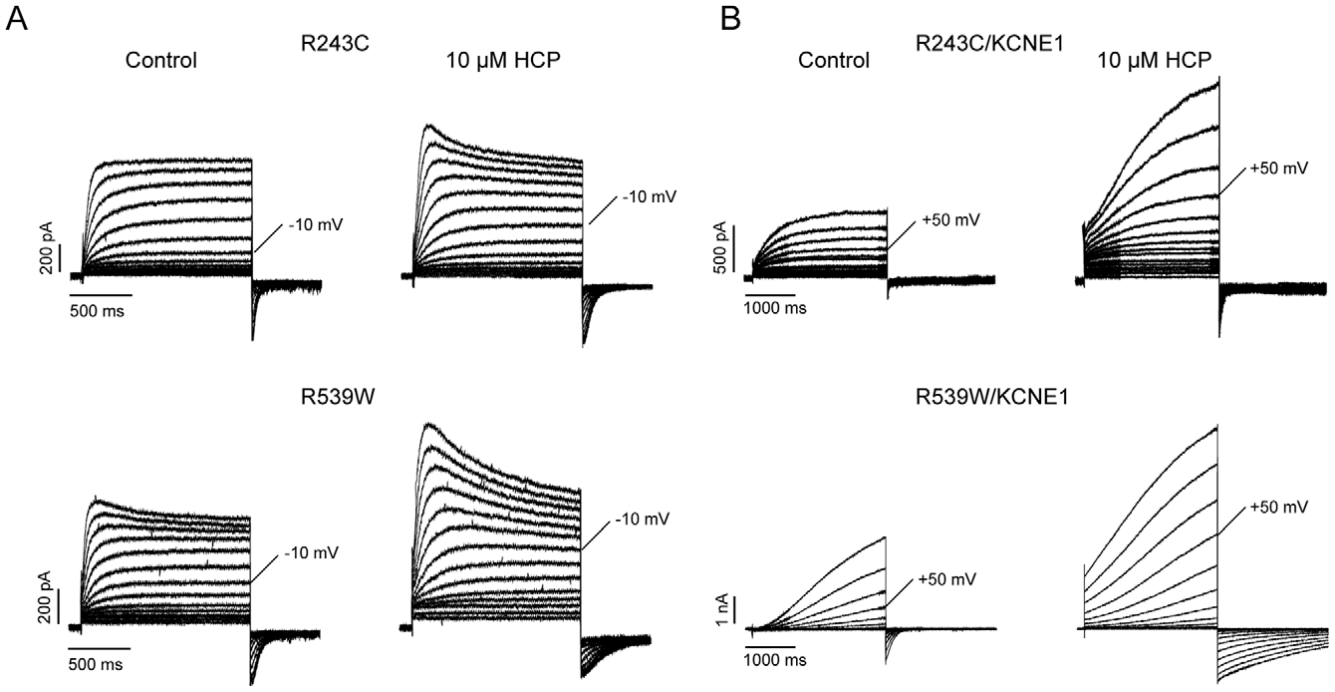
Effects of HCP on two LQTs mutants. **A**. Representative traces of homomeric R243C and R539W with and without 10 μM HCP. **B**. Representative traces of R243C/KCNE1 and R539W/KCNE1 with and without 10 μM HCP.

Mutations in KCNQ1 have been found to cause LQTs [1], [19]. The common phenotype of these mutants is reduction of IKs current, which are commonly thought to be mediated by the KCNQ1/KCNE1 complex [2], [3], as a result of decrease in either channel activity or trafficking efficiency. HCP has exhibited potent effects on both the homomeric KCNQ1 and the KCNQ1/KCNE1 complex; we thus hypothesize its ability to rescue the function reduction of these mutants. We selected and expressed four mutants, R190Q, T587M, R243C and R539W, which are located in different regions of KCNQ1. Among these mutants, R243C and R539W exhibited low but consistent currents, while the others did not exhibit detectable currents in CHO cells. R243 is located near the C-terminal end of S4 transmembrane domain, while R539W is located at the C-terminal tail [20], [21]. Mutations R243C and R539W were thought to impair activation gating or to reduce PIP2 binding affinity, respectively. In the current study, both homomeric mutant channels were significantly potentiated by 10 μM HCP. The potentiation on R243C and R539W were comparable with that on KCNQ1 wild type (Fig. 6 and Table 2). We next tested whether HCP was effective on the mutant/KCNE1 and found that both complexes were also sensitive to 10 μM HCP. The outward currents of R243C/KCNE1 and R539W/KCNE1 were potentiated by 1.44±0.33 (n = 5) and 2.91±0.89 (n = 5) fold, respectively. The effects of HCP on voltage-dependent activation of the mutant channels were also examined and the results are shown in Table 2. Besides R243C/KCNE1, the *G*-*V* curves of all tested mutant channels were left-shifted by 10 μM HCP.

**Table 2:** Effects of HCP on the mutants.

Channel Subtype	*I*/*I*_0_	*ΔV_1_*_/*2*_	n
KCNQ1	1.48±0.13*	−29.92±1.93*	3
KCNQ1/KCNE1	4.47±0.70*	−66.05±6.15*	3
R243C	1.54±0.26*	−22.41±1.03*	5
R243C/KCNE1	1.44±0.33*	+3.90±4.00	7
R539W	1.84±0.21*	−30.83±0.55*	3
R539W/KCNE1	2.91±0.89*	−29.35±2.74*	4

*I*/*I*_0_, *I*, and *I*_0_ are same as being referred in Table 1. To calculate the *I*/*I*_0_, currents were measured at −10 mV for KCNQ1, R243C and R539W. But for KCNQ1/KCNE1, R243C/KCNE1 and R539W/KCNE1, the currents were measured at +50 mV. The concentration of HCP was 10 μM. *p<0.05 versus control.

## Discussion

HCP, also known as Nabac, is the active component of pHisoHex, a prescription drug widely used as an effective antibacterial skin cleanser in the treatment of acne [17]. In previous studies, HCP has been identified as an inhibitor of enoyl-acyl carrier protein reductase and 3CL protease of SARS-CoV [22], [23]. It was also found to attenuate Wnt/β-catenin signaling pathway, which plays important roles in cell proliferation, differentiation and oncogenesis [24]. In the current study, we identified HCP as an activator of KCNQ channels.

### Subtype specificity of HCP

Among all reported activators for KCNQ channels, HCP exhibits unique subtype selectivity. The potentiation on outward current for these homomeric KCNQ channels at −10 mV is: Q1<Q2<Q4. HCP lacks sensitivity to KCNQ3. The well-characterized KCNQ activator, retigabine, lacks sensitivity to KCNQ1. ztz240, another KCNQ activator that is distinct from retigabine and ZnPy, potentiates KCNQ2 and KCNQ4 but does not affect KCNQ1 and KCNQ3. Although ZnPy shows similar selectivity to HCP, *i.e.* potentiates all KCNQ isoforms except KCNQ3, ZnPy lacks sensitivity to the KCNQ1/KCNE1 complex expressed in heterologous system [13]. Here we show HCP exhibited more potent effects on the complex than that on the KCNQ1. The unique subtype selectivity supports HCP may potentiate KCNQ channels through a different mechanism from known activators.

### HCP represents a novel class of KCNQ1/KCNE1 activator

R-L3 potentiates the homomeric KCNQ1 channel at very low concentration, but does not affect the KCNQ1/KCNE1 complex. Interestingly, potentiation effects of R-L3 on the complex seems dependent on the KCNE1 to KCNQ1 ratio. An increased ratio causes noticeable reduction of R-L3 activity [14]. Alanine scanning revealed that R-L3 may interact with specific residues located in the S5 or S6 transmembrane domains of KCNQ1 subunits [25]. Consistently, our previous study showed that residues critical for the potentiation effects by KCNE1 are clustered together in the S6 region in proximity with the critical residues for ZnPy [13]. Different from R-L3 and ZnPy, HCP potentiates both the homomeric KCNQ1 and the KCNQ1/KCNE1 complex. Mefenamic acid, DIDS and PBA were reported KCNQ1/KCNE1 activators [15], [16]. Although all three compounds exhibit potent effects on the KCNQ1/KCNE1 complex, similar to HCP, the phenotypes of the three activators easily differentiate HCP from them. Mefenamic acid or DIDS also cause instantaneous opening of the KCNQ1/KCNE1 complex [26]. However, mefenamic acid and DIDS effects on the homomeric KCNQ1 are much weaker than HCP. They left-shift the *G*-*V* curve and slow deactivation but do not increase overall conductance of homomeric KCNQ1 [15], [27]. Differently, HCP significantly increases the overall conductance of the homomeric KCNQ1 (Fig. 2). PBA potentiates both the KNCQ1 and the KCNQ1/KCNE1. Of interest, PBA exhibits inhibitory effect initially and then gradually potentiation effect on both homomeric KCNQ1 and the KCNQ1/KCNE1 complex. These again argue that PBA and HCP act via different mechanisms [16]. It is of interest that both PBA and HCP have slow off rate; the effects once reaching steady state were difficult to wash off. Taken together, these results suggest that HCP may represent a novel class of KCNQ1/KCNE1 activator.

### A useful tool for development of therapeutics for LQTs

Current therapy for LQTs is inadequate. Because the critical role of the current mediated by KCNQ1/KCNE1 in repolarization of cardiac action potential, augmenting KCNQ1/KCNE1 by small molecule may represent an attractive strategy to treat LQTs. Our study found that 1 μM HCP effectively potentiated native IKs and left-shifted the G-V curve. IKs was activated instantaneously in higher concentrations of HCP, including 3 μM or 10 μM. The effects of HCP on shortening the action potential duration in cardiomyocytes further supports its potential use for development therapeutics for LQTs either as a tool compound or as a lead compound. The slight reduction of the action potential amplitude suggests that HCP may affect other channel(s). Most of LQTs-causing mutations were from KCNQ1 among the identified LQT-associated genes including KCNQ1, HERG,SCN5A, KCNE1 and KCNE2 [4], [5], [19]. These mutations cause reduction in the potassium current, thereby leading to prolongation of cardiac repolarization [7], [28], [29]. Many mutations of LQTs are trafficked to the plasma membrane, suggesting no major folding defects, but reduction of activity [7], [29]. Among the tested mutants, R190Q is a novel identified mutation located at the linker of S2-S3. It is thought to affect the activation gating of KCNQ1/KCNE1. R587M is a trafficking deficient mutation located at the C-terminal end. Loss of function of R190Q and R587M is consistent to previous reports [30], [31]. R243C is a missense mutation located in S4, a transmembrane segment implicated in activation gating of potassium channels [20]. The suppressed current of R243C/KCNE1 is consistent with the notion that the mutation prevents normal channel gating. The additional mutant R539W was identified in patients with a dominantly inherited classical long QT (Romano–Ward) syndrome. R539 is located at the C-terminal tail and shows a reduced PIP2 binding affinity [21]. It has been demonstrated that PIP2 is necessary for the function of various ion channels including KCNQ channels [32], [33]. A recent study showed that the PIP2 affinity of R539W/KCNE1 is reduced [34]. Potentiation of both R243C and R539W suggests HCP is capable of restoring loss-of-function caused by various mutations, either with impaired gating (R243C) or reduced PIP2 affinity (R539W). However, the effects of HCP on the KCNQ1/KCNE1 mutant channels are weaker than that on wild type KCNQ1/KCNE1 channel. For the wild type KCNQ1/KCNE1 channel, 10 μM HCP dramatically left shifts the G-V curve around −66.05±6.15 mV. In contrast, the left shifting for KCNQ1(R539W)/KCNE1 is only −29.35±2.74 mV, and furthermore, in the case of KCNQ1(R243C)/KCNE1, the shifting was negligible. Considering physiological efficiency of an activator of KCNQ channel more relies on its effect on the G-V relationship, the weaker or loss of effects of HCP on the G-V curves of these KCNQ1/KCNE1 mutant channels should be considered as major factors in developing therapeutic for LQTs.

## Conclusions

In conclusion, we identified HCP, the active component of a prescription drug, as a potent KCNQ channel activator with unique subtype selectivity. It exhibits strong potentiation effects on both the homomeric KCNQ1 and the KCNQ1/KCNE1 complex. The distinct pharmacological phenotype and chemical structure of HCP from other reported activators suggest that HCP represents a novel class of KCNQ1/KCNE1 activators. The activity of shortening the action potential duration and the ability to rescuing the loss-of-function of LQTs mutants make HCP a useful tool in development of therapeutics for LQTs.
